# Metazoan parasite fauna of the American mink (*Neogale vison*) in comparison with the closely related European mink (*Mustela lutreola*) in Europe

**DOI:** 10.1007/s00436-025-08543-8

**Published:** 2025-08-18

**Authors:** Anna V. Schantz, Robin Stutz, Anne Steinhoff, Norbert Peter, Sven Klimpel

**Affiliations:** 1 Institute for Ecology, Evolution and Diversity, Goethe-University, Max-Von-Laue-Str. 13, 60438 Frankfurt/ Main, Germany; 2 Senckenberg Biodiversity and Climate Research Centre, Senckenberg Gesellschaft Für Naturforschung, Senckenberganlage 25, 60325 Frankfurt/ Main, Germany

**Keywords:** Parasite diversity, Introduced species, Mink, Ecosystem

## Abstract

The American mink *Neogale vison* is native to North America and was introduced to Europe at the beginning of the twentieth century. Although *N*. *vison* is not yet listed as an invasive species in Europe, it is suspected of having negative impacts on native ecosystems. The native European mink *Mustela lutreola* is considered highly endangered in Europe and the population is continuously declining. It is assumed that the American mink is having an impact on the remaining populations. 50 American mink from Central Europe (Hesse/Germany) were comprehensively examined parasitologically through necropsy and fecal analysis. Further, an extensive literature search on parasitization was done to compare the American and European mink in Europe. Nine parasite species (two ecto- and seven endoparasites) could be identified parasitizing *N*. *vison* in this study. Through necropsy, the highest prevalence was achieved by *Ixodes* ticks with > 65.0%, the most common endoparasite was *Isthmiophiora melis* with 24.0%. *Capillaria putorii* and *C. aerophila* were detected in American mink in Germany for the first time by this investigation. The literature search resulted in the identification of 65 parasite species in twelve European countries for *N*. *vison* and 37 parasite species in six European countries for *M*. *lutreola*. *Neogale vison* is superior in body size, establishing well in Europe, appears to carry a more diverse parasite fauna and seems to be in direct competition to the disappearing European mink.

**Graphical Abstract:**

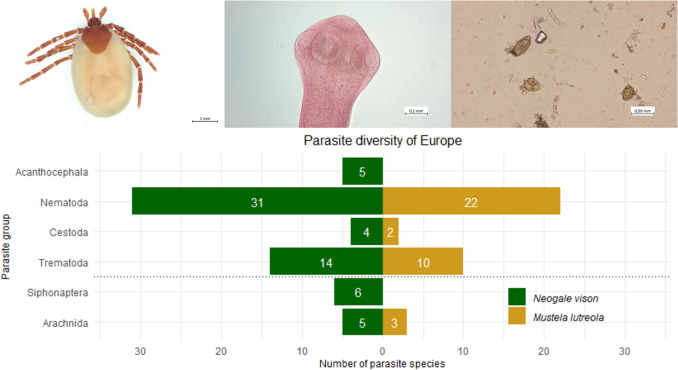

**Supplementary Information:**

The online version contains supplementary material available at 10.1007/s00436-025-08543-8.

## Introduction

The American mink *Neogale vison* (syn.: *Mustela vison*, *Neovison vison*), is native to North America. At the beginning of the twentieth century, it was introduced to the former USSR and Europe, as well as Asia and South America, for the purpose of fur farming and was mainly kept in fur farms (e.g. Dunstone 1993; Lecis et al. 2008). Accidental escapes and deliberate releases have led to the establishment of stable, free-ranging populations within Europe (Lecis et al. 2008; Martínez-Rondán et al. 2017; Vada et al. 2023; Zschille et al. 2012). In Germany, the American mink is a non-native or alien species. The first records of free-ranging American mink were made in Schleswig–Holstein around 1950 (Heidemann 1983), but they are now mainly found in the northern and eastern parts of Germany (Bonesi and Palazon 2007; Zschille et al. 2004). *Neogale vison* is a crepuscular and nocturnal solitary animal, visually it resembles the native European mink *Mustela lutreola*. Both species are mainly found near water and feed opportunistically on small mammals, fish, amphibians, reptiles, birds and their eggs as well as aquatic invertebrates (Hunter and Barrett 2012; Lodé 1993; Maran et al. 1998). Although the American mink is alien to its new range in Europe, it is not yet listed here as an invasive alien species of Union concern (Regulation (EU) No. 1143/2014), on which species with proven negative impacts on native ecosystems are listed. Nevertheless, negative impacts and damages caused by the American mink can be observed. *Neoglae vison* is suspected of causing predation pressure on native, partially endangered and protected prey populations (Barreto et al. 1998; Bonesi and Palazon 2007; Ferreras and Macdonald 1999) and it can also act as a vector of various parasites and pathogens, such as Dunker's muscle fluke (*Alaria alata*), the nematode *Molineus patens*, the Aleutian Mink Disease Virus (AMDV) and as a reservoir host for SarsCoV (e.g. Aguiló-Gisbert et al. 2021; Fournier-Chambrillon et al. 2004; Nugaraitė et al. 2019; Zschille et al. 2004). In contrast *M*. *lutreola* is currently one of the most endangered small carnivores worldwide and only occurs in less than a fifth of its original range (Maran and Henttonen 1995). The IUCN Red List of Threatened Species classifies the European mink as “critically endangered” across Europe (Maran et al. 2012). According to the Rote Liste Zentrum (Meinig et al. 2020), it already has the status “extinct or lost” in Germany. Apart from factors such as habitat loss, hunting and pollution, competition with the American mink also appears to be a reason for the dramatic decline of the European mink (Maran and Henttonen 1995). Since *N*. *vison* occupies similar to almost the same ecological niches as native mustelids, especially those of the European mink, but also of the otter (*Lutra lutra*) or the European polecat (*Mustela putorius*) (Melero et al. 2012; Nugaraitė et al. 2019; Sidorovich et al. 1999), competition for habitat and food arises there. Further consideration of the factor of parasite and disease transmission and spread, caused by the alien American mink, may represent an additional risk factor for the already widely displaced European mink.

The present study supplements the previously known data on the parasite diversity of the American mink *N*. *vison* in Germany. It also summarizes the known parasite fauna of the American and European mink in Europe and thus provides a detailed overview of the parasitization of both species. The compilation of parasitological data on *N*. *vison* and *M*. *lutreola* ensures a well-founded assessment of the zoonotic potential of both species for domestic, farm and wild animals and humans with parasites and other pathogens as well as the protection of endangered prey species and native mustelids (especially the European mink).

## Material & methods

### Sampling

A total of 50 American mink were examined. The animals surveyed came from the Wetterau district (Bad Vilbel and Wöllstadt, Germany). Sampling took place during the permitted hunting season in Hesse (01.09.—28.02.) between the years 2019 and 2023 and was carried out in accordance with the legal hunting practice in Hesse/Germany. The animals to be examined were individually frozen in PE bags at −20 °C until further processing in the laboratory and removed for thawing about 24 h before processing.

### Laboratory work

In order to compare the animals with regard to sex and health status, various morphometric parameters were recorded, the measurements of the examined American mink followed the guidelines of Stubbe and Krapp (1993). The fur was systematically combed to investigate ectoparasitic infestation. Parasite species found were stored in 70% EtOH for further morphological determination. The animals were dissected according to the protocol for the dissection of mammals by Storch and Welsch (2014). After opening the abdominal cavity, the entire organ complex from the tongue to the anus was extracted, the organs were separated from each other and individually divided into smaller parts. Subsequently, individual organs (trachea, lungs, heart, stomach, small intestine, large intestine, kidney, liver, spleen) were opened lengthwise and checked for anomalies and endoparasitic stages. If the skull was intact, it was examined for anomalies and parasite infestation and then prepared. Occurring parasitic individuals were stored in 70% EtOH for further morphological determination.

In addition, fecal samples were taken from the rectum of each examined American mink to investigate them for the presence of further parasitic stages. The samples were frozen at −70 °C until further processing. The feces were examined for parasitic egg and larval stages using the MIFC method (Merthiolate-Iodine-Formaldehyde-Concentration) according to Mehlhorn et al. (1993).

### Species identification, calculations and statistics

All endo- and ectoparasites were identified morphologically. In order to make the external characteristics clearly visible, permanent preparations were made of the parasites according to Klimpel et al. (2019) and identified using the following identification literature: Ticks (Hornok et al. 2021; Lucius et al. 2018; Page and Langton 1996; Petney et al. 2012; Sándor 2017), Digenea (Beaver 1941; Dönges 1973; Hildebrand et al. 2015; Möhl et al. 2009; Nugaraitė et al. 2017; Schmäschke 2014; Zajac and Conboy 2012; Zaleśny et al. 2023), Cestoda (Deplazes et al. 2019; Freeman 1956; Nakao et al. 2013; Šlais 1973), Nematoda (Durette-Desset and Pesson 1987; Kołodziej-Sobocińska et al. 2021; Marroquín-Muciño et al. 2017; Popiołek et al. 2009; Schmäschke 2014; Zajac and Conboy 2012), Acanthocephala (Golvan 1969).

Parasitic egg and larval stages from the fecal examination were photographed and measured during the MIFC (software: ZEISS ZEN Core, version 3.9) and determined based on morphological characteristics (Schmäschke 2014; Zajac and Conboy 2012).

The calculations for parasitic infestation (prevalence P [%], mean intensity mI, maximum intensity maxI, mean abundance mA for parasites from the dissection, prevalence P [%] for results from the MIFC) followed the guidelines of Klimpel et al. (2019).

Analyses of the morphometric data, as well as the creation of figures, were performed using R (R Core Team 2025). Each morphometric variable was divided into male and female subgroups and tested for normal distribution using the Shapiro–Wilk test (α = 0.05; p > 0.05 for normal distribution). Where normal distribution was observed, the homogeneity of variances between the two subgroups was tested using the Levene test (α = 0.05; p > 0.05 for variance homogeneity). Where there was normal distribution but not variance homogeneity, the two-tailed Welch t-test was used. If at least one of the two groups was not normally distributed, a two-tailed Mann–Whitney-U test for non-parametric samples was performed. A significance level of α = 0.05 was applied to all p-values. Due to multiple testing, Bonferroni correction was applied to all p-values to counteract alpha error accumulation. Results of statistical analysis are given in Table S1 in the supplementary information.

### Literature research

A comprehensive literature search was carried out to collect data on parasitization, disease transmission and disease spreading to compare the two mustelid species *N*. *vison* and *M*. *lutreola* within Europe. Available publications relating to the mentioned topics and at least one of the two species in Europe were used, regardless of the year of publication. The keywords “mink, American mink, *Neogale vison*, *Neovison vison*, *Mustela vison*, European mink, *Mustela lutreola*” combined with “parasite(s), parasite fauna, parasite diversity, parasite occurrence, endoparasites, ectoparasites, infection, infestation, disease(s), transmission, zoonoses,” were specified for the literature research via the common literature search engines, mostly Google Scholar. Based on the data collected in this context, a comparative overview table was compiled (Tab. 2), which was provided with the respective literature references (Abalihin et al. 2019; Anisimova 2004; Anisimova and Poloz 2010; Christian 2010; Fairley 1980; Fournier-Chambrillon et al. 2018; Gilot and Aubert 1985; Górski et al. 2006; Harrington et al. 2012; Hawkins et al. 2010a, b; Heddergott et al. 2016; Hurníková et al. 2016; Klockiewicz et al. 2023; Kołodziej‐Sobocińska et al. 2018, 2021; Kondzior et al. 2020; Kornyushin et al. 2011, 2022; Korol et al. 2016; Larsen et al. 2018; Lemming et al. 2020; Martínez-Rondán et al. 2017; Maslennikova and Strelnikov 2020; Nugaraitė et al. 2014, 2019; Page and Langton 1996; Palomar et al. 2025; Refojos et al. 2006; Romanov 1964; Romashova et al 2014; Sherrard-Smith et al. 2015; Shimalov and Shimalov 2001a; Simpson et al. 2005; Skov et al. 2008; Sleeman and Smiddy 2004; Smiddy and Sleeman 1998; Tăbăran et al. 2013; Torres et al. 2003, 2006, 2008, 2016; Trebbien et al. 2014; Troitskaya 1960, 1967, 1971; Varodi et al. 2017; Zschille et al. 2004).

## Results

### Parasite examination data

Of the 50 American mink examined, 36 were male and 14 were female. All mean values of morphometric data, except ear length, were significantly higher (α = 0.05, Bonferroni-adjusted) for males than for females. No anomalies were found in the examined animals. Morphometric data are available in Table S1.

**Fig. 1:**
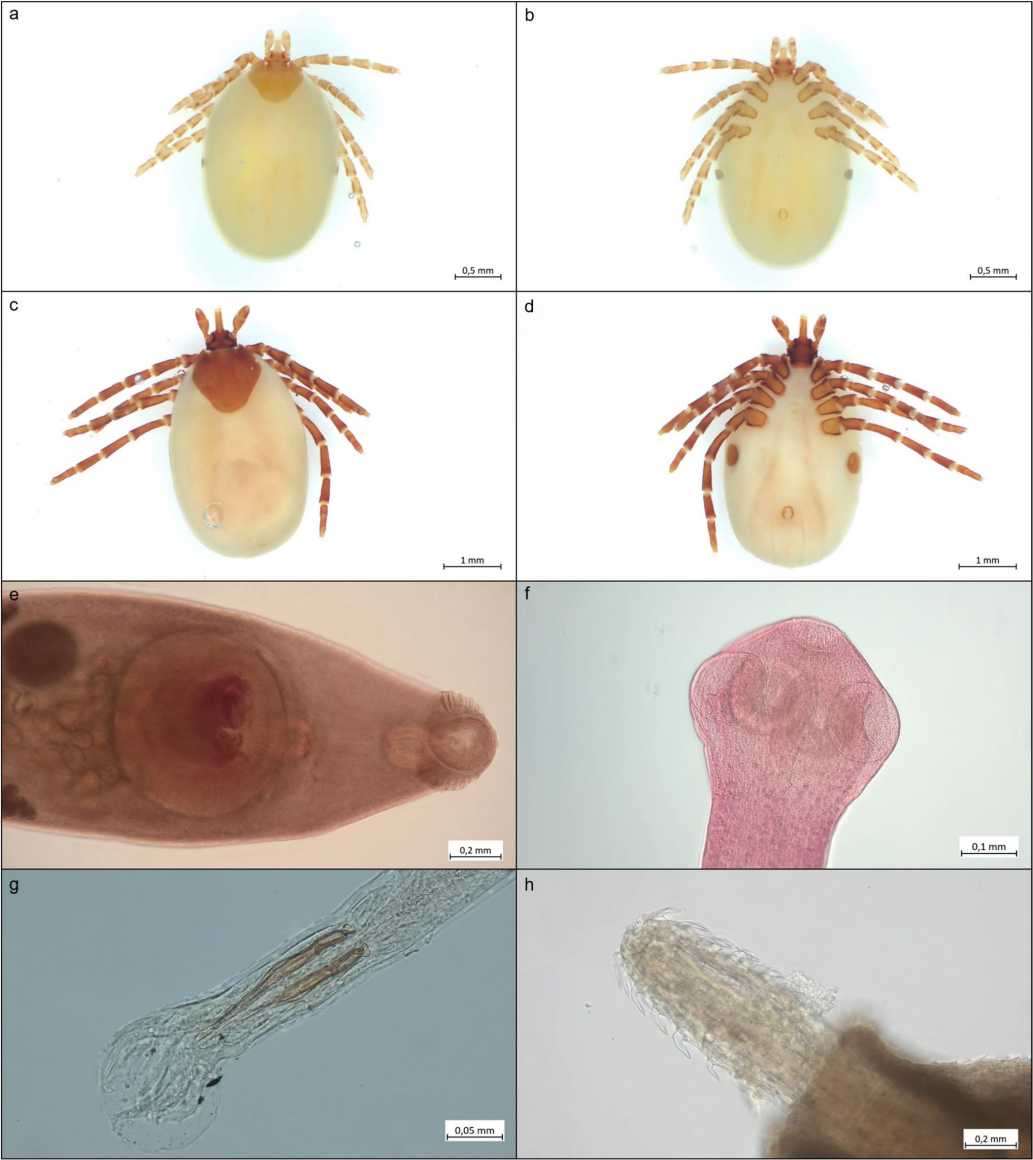
Ecto- and endoparasites found in examined American mink via parasitological dissection. a: Dorsal view of female *Ixodes canisuga*; b: Ventral view of female *I*. *canisuga*; c: Dorsal view of female *I*. *hexagonus*; d: Ventral view of female *I*. *hexagonus*; e: *Isthmiophora melis*; f: *Versteria mustelae*; g: *Molineus patens*; h: *Acanthocephalus lucii*

Based on the external examination, a total of two parasites could be identified to species level. The ectoparasites found were determined to be *Ixodes canisuga* (P = 32.0%, mI = 2.4, maxI = 8.0, mA = 0.8) and *I*. *hexagonus* (P = 42.0%, mI = 3.6, maxI = 11.0, mA = 1.5) (Fig. 1a-d). Furthermore, individuals were found that were assigned to the genus *Ixodes* but could not be morphologically determined to species level due to the juvenile stage of development (P = 66.0%, mI = 24.9, maxI = 260.0, mA = 16.4) (Tab. 1).

**Table 1:** Parasitological calculations of the examined American mink, Prevalence (P [%]), mean Intensity (mI), maximum Intensity (maxI) and mean Abundance (mA) were calculated for males (M, n = 36), females (F, n = 14) and all examined animals (M + F, N = 50)

Male (M)/Female (F)	Value	Ectoparasites			Endoparasites						
		*Ixodes canisuga*	*Ixodes hexagonus*	*Ixodes* spp.	Alaria alata *	*Isthmiophora melis*	*Versteria mustelae*	Capillaria putorii *	Capillaria aerophila *	*Molineus patens*	*Acantocephalus lucii*
M	P [%]	33.3	50.0	66.7	8.3	22.2	2.8	2.8	2.8	2.8	5.6
	mI	2.7	3.9	31.6	-	14.9	1.0	-	-	2.0	2.0
	maxI	8.0	11.0	260.0	-	25.0	1.0	-	-	2.0	2.0
	mA	0.9	2.0	21.1	-	3.3	0.1	-	-	0.1	0.1
F	P [%]	28.6	21.4	64.3	-	28.6	-	-	-	-	-
	mI	1.8	1.3	7.1	-	14.5	-	-	-	-	-
	maxI	4.0	2.0	22.0	-	45.0	-	-	-	-	-
	mA	0.5	0.3	4.6	-	4.1	-	-	-	-	-
M + F	P [%]	32.0	42.0	66.0	6.0	24.0	2.0	2.0	2.0	2.0	4.0
	mI	2.4	3.6	24.9	-	14.8	1.0	-	-	2.0	2.0
	maxI	8.0	11.0	260.0	-	45.0	1.0	-	-	2.0	2.0
	mA	0.8	1.5	16.4	-	3.5	<0.1	-	-	<0.1	0.1

^*^ Only found in fecal analysis (MIFC)

During the organ dissection, endoparasites of the parasite groups Digenea, Cestoda, Nematoda and Acanthocephala could be identified, all of them were located in the small intestine. The most frequently occurring endoparasite species was the digenea *Isthmiophora melis* (Fig. 1e) with a prevalence (P) of 24.0%, a mean intensity (mI) of 14.8 and a mean abundance (mA) of 3.5. The maximum intensity (maxI) was 45.0 individuals of *I*. *melis* in a female American mink. The cestode species *Versteria mustelae* (Fig. 1f) and the nematode species *Molineus patens* (Fig. 1g) were each detected in only one dissected animal (P = 2.0%). *Acanthocephalus lucii* (Fig. 1h) was the only acanthocephalan species and occurred twice, each time with two individuals in each *N*. *vison*. This resulted in a prevalence (P) of 4.0%, a mean and maximum intensity (mI & maxI) of 2.0 and a mean abundance (mA) of 0.1 for all American mink examined (Tab. 1).

**Fig. 2:**
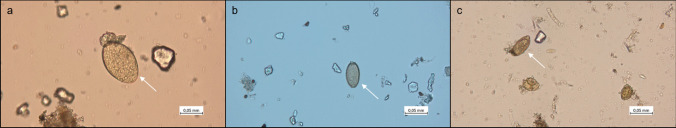
Parasite eggs found in examined American mink via fecal investigation. a: *Alaria alata* egg; b: *Capillaria putorii* egg; c: *Capillaria aerophila* egg

Three parasite species were detected in the fecal samples (Tab. 1). With a prevalence (P) of 6.0% in the fecal samples of all American mink examined, eggs of the fluke *Alaria alata* (Fig. 2a) were detected. In addition, eggs of the two hairworm species *Capillaria putorii* (Fig. 2b) and *C. aerophila* (Fig. 2c) were identified in 2.0% of the animals examined.

### Literature research and comparison

The literature search resulted in the verification of 65 parasite species for the American mink and 37 parasite species for the European mink in 48 publications. The American mink shows a more diverse parasite fauna than the European mink, 30 parasite species have been detected in Europe in both mustelid species (Tab. 2). In Europe, there is evidence of parasite findings in the American mink in twelve countries and for the European mink in six countries. For *M*. *lutreola* there are studies from 1964 to 2025, while for *N*. *vison* there are parasitological studies in Europe from 1960 to the present.

**Table 2:** Comparison of parasite findings in *N*. *vison* and *M*. *lutreola* in Europe based on literature research and the present study; if available, the associated parasite prevalence P [%] was indicated

		**Parasite species**	**Country**	**American mink (*Neogale vison*)**	**European mink (*Mustela lutreola*)**
**Ectoparasites**	**Arachnida**	*Ixodes acuminatus*^p^	England and Wales	**P = 0.1%** Page and Langton (1996)	
			Spain	Palomar et al. (2025); Refojos et al. (2006)	Palomar et al. (2025); Refojos et al. (2006)
		*Ixodes canisuga*	Germany	Christian (2010); **P = 32.0%** [present study]	
			England and Wales	**P = 2.5%** Page and Langton (1996)	
		*Ixodes hexagonus*^p^	England and Wales	**P = 40.0%** Page and Langton (1996)	
			France		Gilot and Aubert (1985)
			Germany	**P = 49.0%** Zschille et al. (2004); Christian (2010); **P = 42.0%** [present study]	
			Ireland	Fairley (1980)	
			Spain	Palomar et al. (2025); Refojos et al. (2006)	Palomar et al. (2025); Refojos et al. (2006)
		*Ixodes ricinus*^p^	England and Wales	**P = 0.1%** Page and Langton (1996)	
			Germany	**P = 3.3%** Zschille et al. (2004); Christian (2010)	
		*Ixodes rugicollis*	Germany	**P = 3.3%** Zschille et al. (2004); Christian (2010)	
		*Ricicephalus sanguineus s.l.*^p^	Spain		Palomar et al. (2025)
	**Siphonaptera**	*Ceratophyllus sciurorum* (syn. *Monopsyllus sciurorum*)^f^	Denmark	Larsen et al. (2018); Trebbien (2014)	
		*Ctenophthalmus nobilis vulgaris*	Ireland	Fairley (1980)	
		*Doratopsylla dasycnema dasycnema*	Ireland	Smiddy and Sleeman (1998)	
		*Hystrichopsylla talpae talpae*	Ireland	Smiddy and Sleeman (1998)	
		*Nosopsyllus fasciatus*^p^	Ireland	Fairley (1980); Sleeman and Smiddy (2004); Smiddy and Sleeman (1998)	
		*Typhloceras poppei*	Ireland	Fairley (1980)	
**Endoparasites**	**Trematoda**	*Alaria alata*^p^	Belarus	**P = 2.8%** Anisimova and Poloz (2010); **P = 6.0%** Shimalov and Shimalov (2001a)	**P = 4.0%** Anisimova (2004)
			Germany	**P = 10.6%** Zschille et al. (2004); **P = 6.00%** [present study]	
			Lithuania	**P = 7.6%** Nugaraité et al. (2019)	
			Poland	**P = 12.5%** Górski et al. (2006)	
			Romania		**P = 100.0%** Tăbăran et al. (2013)
			Russia	**P = 43.6–51.0%** Maslennikova and Strelnikov (2020)	
		*Apophallus donicus* (syn. *Rossicotrema donicum*)^p^	Belarus	**P = 0.8%** Anisimova and Poloz (2010); **P = 6.0%** Shimalov and Shimalov (2001a)	**P = 4.0%** Anisimova (2004)
			Spain		**P = 3.6%** Torres et al. (2003)
			Ukraine	**P = 38.5%** Kornyushin et al. (2022); **P = 30.7%** Korol et al. (2016)	
		*Echinochasmus perfoliatus*	Russia	**P = 54.2%** Abalihin et al. (2019)	**P = 20.0%** Abalihin et al. (2019)
			Ukraine	**P = 38.5%** Kornyushin et al. (2022); **P = 15.4%** Korol et al. (2016)	
		*Echinostoma jurini *(syn*. Echinostoma sisjakowi; Echinoparyphium sisjakowi*)	Russia	Troitskaya (1967)	
		*Euryhelmis squamula*	France	**P = 26.3%** Torres et al. (2008)	**P = 15.4%** Torres et al. (2008)
			Spain	**P = 2.0–3.2%** Torres et al. (2003)	**P = 17.9%** Torres et al. (2003)
		*Isthmiophora melis* (syn. *Euparyphium melis*)^p^	Belarus	**P = 27.1%** Anisimova and Poloz (2010); **P = 20.0%** Shimalov and Shimalov (2001a)	**P = 28.0–41.9%** Anisimova (2004)
			France	**P = 2.6%** Torres et al. (2008)	**P = 2.6%** Torres et al. (2008)
			Germany	**P = 56.0%** Zschille et al. (2004); **P = 24.0%** [present study]	
			Lithuania	**P = 66.0%** Nugaraité et al. (2014); **P = 70.0–77.0%** Nugaraité et al. (2019)	
			Poland	Klockiewicz et al. (2023); Kołodziej-Sobocińska (2018)	
			Russia	**P = 45.6–56.4%** Maslennikova and Strelnikov (2020); **P = 60.0%** Romanov (1964); **P = 67.2%** Romashova et al. (2014); Troitskaya (1960)	**P = 20.0%** Romanov (1964)
			Ukraine	Kornyushin et al. (2022); **P = 61.5%** Korol et al. (2016)	
		*Isthmiophora inerme*	Belarus	**P = 5.7%** Anisimova and Poloz (2010)	
		*Mammorhcipedum isostomum*	Russia	**P = 5.1–5.7%** Maslennikova and Strelnikov (2020); **P = 17.3%** Romashova et al. (2014); Troitskaya (1971)	
		*Metorchis bilis* (syn. *Metorchis albidus*)^p^	Belarus	**P = 1.6%** Anisimova and Poloz (2010); **P = 8.0%** Shimalov and Shimalov (2001a)	**P = 4.0%** Anisimova (2004)
			Germany	**P = 6.0%** Zschille et al. (2004)	
			Russia	Maslennikova and Strelnikov (2020); **P = 17.3%** Romashova et al. (2014)	
			Spain		**P = 32.1%** Torres et al. (2003)
		*Nanophyetus salminicola*^p^	Russia		**P = 8.0%** Abalihin et al. (2019)
		*Opisthorchis felineus*^p^	Belarus	**P = 2.4%** Anisimova and Poloz (2010); **P = 4.0%** Shimalov and Shimalov (2001a)	
			Russia	**P = 33.4%** Romashova et al. (2014)	
		*Paragonimus westermani*^p^	Russia	**P = 0.9%** Abalihin et al. (2019)	**P = 4.0%** Abalihin et al. (2019)
		*Pseudamphistomum truncatum*^p^	Belarus	**P = 1.6%** Anisimova and Poloz (2010); **P = 6.0%** Shimalov and Shimalov (2001a)	**P = 4.0%** Anisimova (2004)
			Denmark	**P = 100.0%** Skov et al. (2008)	
			England	Harrington et al. (2012); **P = 42.9%** Simpson et al. (2005)	
			England and Wales	**P = 33.0%** Sherrard-Smith et al. (2015)	
			France		**P = 22.9%** Torres et al. (2008)
			Ireland	**P = 3.5%** Hawkins et al. (2010a)	
			Lithuania	**P = 17.9–30.0%** Nugaraité et al. (2019)	
			Russia	**P = 67.2%** Romashova et al. (2014); Troitskaya (1960), (1967)	
			Spain		**P = 3.6%** Torres et al. (2003)
			Ukraine	Kornyushin et al. (2022); **P = 30.8%** Korol et al. (2016)	
		*Strigea strigis*	Lithuania	**P = 33.0%** Nugaraité et al. (2014); **P = 28.2–30.0%** Nugaraité et al. (2019)	
		*Troglotrema acutum*	France	**P = 33.3%** Torres et al. (2008)	**P = 2.3%** Torres et al. (2008)
			Spain	**P = 2.0%** Martinez-Rondan et al. (2017); **P = 30.4%** Torres et al. (2006)	
	**Cestoda**	*Mesocestoides lineatus*^p^	Germany	**P = 1.5%** Zschille et al. (2004)	
			Russia	**P = 1.9%** Abalihin et al. (2019)	
		*Spirometra erinaceieuropaei*^p^	Belarus	**P = 50.6%** Anisimova and Poloz (2010); **P = 10.0%** Shimalov and Shimalov (2001a)	**P = 58.0–88.0%** Anisimova (2004)
			Poland	**P = 75.0%** Kondzior et al. (2020)	
			Ukraine	Kornyushin et al. (2011); **P = 69.2%** Kornyushin et al. (2022)	
		*Taenia martis*^p^	Germany	**P = 6.0%** Zschille et al. (2004)	
			Lithuania	**P = 2.5%** Nugaraité et al. (2019)	
			Spain	**P = 2.0%** Torres et al. (2003)	
		*Versteria mustelae* (syn. *Taenia mustelae; T. tenuicollis*)	Belarus	**P = 0.8%** Anisimova and Poloz (2010); **P = 4.0%** Shimalov and Shimalov (2001a)	**P = 8.0%** Anisimova (2004)
			Germany	**P = 4.5%** Zschille et al. (2004); **P = 2.0%** [present study]	
			Poland	Klockiewicz et al. (2023)	
			Russia	Maslennikova and Strelnikov (2020)	
			Spain	**P = 3.2%** Torres et al. (2003)	**P = 100.0%** Fournier-Chambrillon et al. (2018)
	**Nematoda**	*Aelurostrongylus pridhami*	Spain		**P = 7.1%** Torres et al. (2003)
		*Ancylostoma caninum*^p^	Russia	**P = 0.9%** Abalihin et al. (2019)	
		*Angiostrongylus daskalovi*	Spain	**P = 6.0%** Martinez-Rondan et al. (2017)	
		*Angiostrongylus vasorum*	Denmark	**P = 0.8%** Lemming et al. (2020)	
		*Aonchotheca annulosa*	Spain	**P = 8.0%** Martinez-Rondan et al. (2017)	
		*Ascaris columnaris*	Russia	**P = 17.3%** Romashova et al. (2014)	
		*Baylisascaris devosi *(syn.* Ascaris devosi*)	Belarus	**P = 2.2%** Anisimova and Poloz (2010); **P = 4.0%** Shimalov & Shimalov (2001a)	**P = 8.0%** Anisimova (2004)
		*Capillaria aerophila* (syn. *Eucoleus aerophilus; Thominx aerophilus*)^p^	Belarus	**P = 2.3%** Anisimova and Poloz (2010)	**P = 3.2%** Anisimova (2004)
			France		**P = 32.5%** Torres et al. (2008)
			Germany	**P = 2.0%** [present study]	
			Lithuania	**P = 11.00** Nugaraité et al. (2014); **P = 10.0–15.3%** Nugaraité et al. (2019)	
			Russia	**P = 6.5%** Abalihin et al. (2019); Maslennikova and Strelnikov (2020)	**P = 16.0%** Abalihin et al. (2019)
		*Capillaria mucronata* (syn. *Aonchotheca mucronata; Pearsonema mucronata*)	Belarus	**P = 49.6%** Anisimova and Poloz (2010); **P = 30.0%** Shimalov and Shimalov (2001a)	**P = 20.0–28.0%** Anisimova (2004)
			Russia	**P = 44.5–48.7%** Maslennikova and Strelnikov (2020); **P = 40.0%** Romanov (1964); **P = 33.4%** Romashova et al. (2014); Troitskaya (1967)	
			Ukraine	Kornyushin et al. (2022); **P = 38.5%** Varodi et al. (2017)	
		*Capillaria mustelorum°*	Belarus		**P = 3.2–24.0%** Anisimova (2004)
		*Capillaria plica (*syn.* Pearsonema plica)*	France	**P = 2.7%** Torres et al. (2008)	**P = 13.3%** Torres et al. (2008)
		*Capillaria putorii (*syn.* Aonchotheca putorii)*	Belarus	**P = 36.3%** Anisimova and Poloz (2010); **P = 20.0%** Shimalov & Shimalov (2001a)	**P = 6.4–20.0%** Anisimova (2004)
			France	**P = 18.4%** Torres et al. (2008)	**P = 15.0%** Torres et al. (2008)
			Germany	**P = 2.0%** [present study]	
			Lithuania	**P = 33.0%** Nugaraité et al. (2014); **P = 10.6–22.1%** Nugaraité et al. (2019)	
			Poland	Kołodziej-Sobocinska et al. (2018), (2021)	
			Russia	**P = 2.8%** Abalihin et al. (2019); **P = 71.8–81.0%** Maslennikova and Strelnikov (2020); Romanov (1964); **P = 33.4%** Romashova et al. (2014); Troitskaya (1960), (1967)	**P = 8.0%** Abalihin et al. (2019); **P = 20.0%** Romanov (1964)
			Spain	**P = 54.0%** Martinez-Rondan et al. (2017); **P = 12.0–25.8%** Torres et al. (2003)	**P = 7.1%** Torres et al. (2003)
			Ukraine	Kornyushin et al. (2022); **P = 23.1%** Varodi et al. (2017)	
		*Crenosoma melesi*	Spain	**P = 10.0%** Martinez-Rondan et al. (2017); **P = 2.0%** Torres et al. (2003)	
		*Crenosoma petrovi*	Belarus	**P = 0.8%** Anisimova and Poloz (2010);	
			Russia	**P = 4.6%** Abalihin et al. (2019)	**P = 12.0%** Abalihin et al. (2019)
		*Crenosoma schachmatovae*	Lithuania	**P = 22.0%** Nugaraité et al. (2014); **P = 10.2–15.0%** Nugaraité et al. (2019)	
		*Crenosoma taiga*	Belarus	**P = 3.9%** Anisimova and Poloz (2010); **P = 4.0%** Shimalov and Shimalov (2001a)	**P = 3.2%** Anisimova (2004)
			Russia	**P = 28.9%** Abalihin et al. (2019); **P = 4.9–5.1%** Maslennikova and Strelnikov (2020)	**P = 8.0%** Abalihin et al. (2019)
		*Crenosoma vulpis*	Denmark	**P = 5.7%** Lemming et al. (2020)	
			Russia	**P = 3.7%** Abalihin et al. (2019)	**P = 4.0%** Abalihin et al. (2019)
		*Filaroides osleri (*syn. *F. bronchialis)*	Russia	Troitskaya (1960)	
		*Filaroides martis*	Belarus	**P = 2.3%** Anisimova and Poloz (2010); **P = 8.0%** Shimalov and Shimalov (2001a)	**P = 16.0–24.0%** Anisimova (2004)
			Germany	**P = 3.0%** Zschille et al. (2004)	
			France		**P = 18.4%** Torres et al. (2008)
			Russia	**P = 1.8%** Abalihin et al. (2019); Maslennikova and Strelnikov (2020); **P = 50.0%** Romanov (1964); Troitskaya (1967)	**P = 4.0%** Abalihin et al. (2019); **P = 100.0%** Romanov (1964); Troitskaya (1967)
		*Filaria martis*	Spain		**P = 12.0%** Torres et al. (2016)
		*Gnathostoma spinigerum*	Ukraine		Varodi et al. (2017)
		*Molineus patens*	Belarus	**P = 3.8%** Anisimova and Poloz (2010); **P = 8.0%** Shimalov and Shimalov (2001a)	**P = 12.0%** Anisimova (2004)
			France	**P = 50.0%** Torres et al. (2008)	**P = 84.6%** Torres et al. (2008)
			Germany	**P = 15.2%** Zschille et al. (2004); **P = 2.0%** [present study]	
			Lithuania	**P = 33.0%** Nugaraité et al. (2014); **P = 12.8–20.0%** Nugaraité et al. (2019)	
			Poland	Kołodziej-Sobocinska et al. (2021)	
			Russia	Maslennikova and Strelnikov (2020)	
			Spain	**P = 68.0%** Martinez-Rondan et al. (2017); **P = 9.7–36.0%** Torres et al. (2003)	**P = 100.0%** Fournier-Chambrillon et al. (2018); **P = 53.6%** Torres et al. (2003)
			Ukraine	Varodi et al. (2017)	
		*Mustelivingylus skrjabini*	Belarus	**P = 3.2%** Anisimova and Poloz (2010); **P = 4.0%** Shimalov and Shimalov (2001a)	
			Russia	Maslennikova and Strelnikov (2020); Romanov (1964)	**P = 60.0%** Romanov (1964)
		*Oswaldocruzia filiformis*	Russia	Maslennikova and Strelnikov (2020)	
		*Skrjabingylus nasicola*	Belarus	**P = 11.7%** Anisimova and Poloz (2010); **P = 16.0%** Shimalov and Shimalov (2001a) [49]	**P = 13.0–32.0%** Anisimova (2004)
			England	Harrington et al. (2012)	
			France	**P = 41.7%** Torres et al. (2008)	**P = 54.5%** Torres et al. (2008)
			Germany	**P = 44.4–62.9%** Heddergott et al. (2016)	
			Ireland	**P = 21.0%** Hawkins (2010b)	
			Russia	**P = 2.8%** Abalihin et al. (2019); **P = 8.7–10.3%** Maslennikova and Strelnikov (2020); **P = 40.0%** Romanov (1964); Troitskaya (1967)	**P = 100.0%** Romanov (1964); Troitskaya (1967)
			Spain	**P = 0.5–8.7%** Torres et al. (2006)	**P = 100.0%** Fournier-Chambrillon et al. (2018)
			Ukraine	Varodi et al. (2017)	
		*Skrjabingylus petrowi*	Russia	Maslennikova and Strelnikov (2020)	
		*Soboliphyme baturini*	Russia		Troitskaya (1967)
		*Sobolevingylus petrowi*	Russia	Maslennikova and Strelnikov (2020); Romanov (1964)	**P = 40.0%** Romanov (1964)
		*Spirocera lupi*	Belarus	**P = 0.8%** Anisimova and Poloz (2010)	
		*Strongyloides martes*	Belarus	**P = 0.8%** Anisimova and Poloz (2010); **P = 2.0%** Shimalov and Shimalov (2001a)	**P = 4.0%** Anisimova (2004)
			Germany	**P = 1.5%** Zschille et al. (2004)	
			Russia	Maslennikova and Strelnikov (2020)	
		*Strongyloides mustelorum*	France	**P = 2.6%** Torres et al. (2008)	**P = 35.9%** Torres et al. (2008)
			Spain		**P = 46.4%** Torres et al. (2003)
		*Trichinella britovi*^p^	Poland	**P = 55.6%** Hurnikova et al. (2016)	
		*Trichinella pseudospiralis*^p^	Poland	Hurnikova et al. (2016)	
			Russia	**P = 0.9%** Abalihin et al. (2019)	
		*Trichinella spiralis*^p^	Belarus	**P = 9.2%** Anisimova and Poloz (2010)	**P = 6.4%** Anisimova (2004)
			Poland	Hurnikova et al. (2016)	
			Russia	**P = 2.8%** Abalihin et al. (2019)	**P = 4.0%** Abalihin et al. (2019)
			Ukraine		Varodi et al. (2017)
		*Uncinaria criniformis*	Germany	**P = 10.6%** Zschille et al. (2004)	
		*Uncinaria stenocephala*^p^	Russia	**P = 18.6%** Abalihin et al. (2019)	**P = 28.0%** Abalihin et al. (2019)
	**Acanthocephala**	*Acanthocephalus anguillae*	Germany	**P = 4.5%** Zschille et al. (2004)	
		*Acanthocephalus lucii*	Germany	**P = 1.5%** Zschille et al. (2004); **P = 4.00%** [present study]	
		*Corynosoma strumosum*	Belarus	**P = 0.8%** Anisimova and Poloz (2010); **P = 4.0%** Shimalov & Shimalov (2001a)	
		*Centrorhynchus ninnii*	Spain	**P = 2.0%** Torres et al. (2003)	
		*Pomphorhynchus laevis*	Germany	**P = 1.5%** Zschille et al. (2004)	

^f^ farm animal

^p^ human pathogenic

° According to Deplazes et al. (2021) a synonym for *Capillaria putorii*

## Discussion

### *Neogale vison* sampled from Germany

The two tick species *I*. *canisuga* and *I*. *hexagonus* were identified as ectoparasites (Fig. 1a-d). The dog tick *I. canisuga* is a typical parasite of the red fox *Vulpes vulpes* and can therefore be detected in its distribution area. The hedgehog tick *I*. *hexagonus* is one of the most common tick species in Europe and its main host is the European hedgehog *Erinaceus europaeus*. Both tick species are widespread throughout Europe and also parasitize numerous other carnivores, including mustelids like *N*. *vison* (Page and Langton 1996; Petney et al. 2012; Refojos et al. 2006; Sándor 2017; Zschille et al. 2004). Due to the low host specificity of both tick species, it is in accordance with expectations that they could be detected in this study.

Four species were identified as endoparasites by examining the internal organs (Fig. 1e-h). *Isthmiophora melis* (Fig. 1e) was the most prevalent endoparasite species with P = 24.0%. It occurs in Europe, Asia and North America, where it is a common parasite of the small intestine of *N*. *vison*, as well as of many other carnivores such as raccoons (*Pocyon lotor*), raccoon dogs (*Nyctereutes procyonoides*) and red foxes (*Vulpes vulpes*) (e.g. Klockiewicz et al. 2023; Kornyushin et al. 2022; Nugaraitė et al. 2014, 2019; Peter et al. 2023, 2024; Schantz et al. 2023; Shimalov and Shimalov 2001a; Torres et al. 2008; Zschille et al. 2004). The life cycle of this Digenea is characterized by two intermediate hosts. The great pond snail *Lymnea stagnalis* acts as the obligate first intermediate host (Faltýnková et al. 2007; Radev et al. 2009), which is infected by free-swimming ciliated larvae (miracidia) that have previously undergone embryogenesis in eggs in the environment (Dönges 1973). The eggs are excreted by the definitive hosts and can survive for several months even at low temperatures, which can contribute to the maintenance of the parasite population (Dönges 1973). After infection of *L*. *stagnalis*, the miracidia develop via sporocysts and several generations of mother and daughter redia into cercariae, which are subsequently expelled (Dönges et al. 1969). Various species of amphibians (e.g. larvae of *Rana temporaria*, *Bufo bufo*, *Bombina variegata* and *Alytes obstetricans* in laboratory experiments (Dönges 1967, 1968)) as well as various species of freshwater fish act as second intermediate hosts (Radev et al. 2009). In these second intermediate hosts, development into the metacercarial stage, which is infectious for the definitive hosts, takes place (Dönges 1967, 1968; Lucius et al. 2018). The detection of this parasite species therefore also allows conclusions to be drawn about the diet of the animals examined and reflects the aquatic lifestyle and diet of *N*. *vison*.

The tapeworm species *V*. *mustelae* (Fig. 1f) was detected in one examined American mink. This up to 10 cm long cestode is a parasite that typically infests mustelids as definitive hosts, especially those of the genera *Martes* and *Mustela*. Rodents, lagomorphs and insectivores (e.g. of the genus *Sorex*) have been described as suitable intermediate hosts (Loos-Frank 2000). In the intermediate hosts, the 0.8—2.0 mm larvae of *V*. *mustelae* are found in cysts between 1–3 mm in size, primarily on or in the liver, but the lungs, spleen, kidneys, heart and brain can also be infested (Loos-Frank 2000; McKeever and Henry 1971). Since these small mammals belong to the prey spectrum of the definitive hosts, the parasites are thus ingested (e.g. Deplazes et al. 2019; Freeman 1956; Langham et al. 1990; Loos-Frank 2000; Lucius et al. 2018; Mahrt and Chai 1972). Also known primarily under the synonyms *Taenia mustelea* or *T*. *tenuicollis*, the new genus *Versteria* was proposed by Nakao et al. (2013) to classify this tapeworm, as this species appears to be more closely related to the genus *Echinococcus* than to other species of the genus *Taenia*. *Versteria mustelea* is already known for parasitizing American mink in Europe (Tab. 2).

Two specimens of the nematode species *M*. *patens* (Fig. 1g) were isolated from a male American mink. *Molineus patens* is a well-known parasite in mustelids (e.g. ermine (*Mustela erminea*), weasel (*M*. *nivalis*), American mink (*N*. *vison*), badger (*Meles meles*)), but also parasitizes for example in raccoon dogs (*N. procyonoides*), raccoons (*P. lotor*) or foxes (*V. vulpes*) in Europe (Kołodziej-Sobocińska et al. 2021; Martínez-Rondán et al. 2017; Nugaraitė et al. 2014, 2019; Peter et al. 2023; Popiołek et al. 2009; Shimalov and Shimalov 2001a, b; Torres et al. 2003, 2008; Zschille et al. 2004). Male nematodes of this species reach a length between 5–7 mm, while females can grow to over 11 mm (Leiper 1936). A detailed description of the life cycle of *M*. *patens* has not yet been described, but it can be assumed that species of the genus *Molineus* are subject to a direct life cycle in which the host can become infected by oral or percutaneous intake of the larvae, as described for *M*. *barbatus*, for example (Gupta 1961, 1963). Female *M*. *patens* lay eggs in the host's intestine, which are excreted in the host's feces and thus enter the environment, where embryonation takes place. The third larval stage (L3 larva) is the infective stage for the definitive host (Gupta 1961, 1963; Pavlovic et al. 2021; Peter et al. 2023; Suchentrunk and Sattmann 1994).

Finally, the acanthocephalan species *A*. *lucii* (Fig. 1h) was identified. It has already been described in American Mink in Germany by Zschille et al. (2004). The adult stage of this acanthocephalid parasitizes in the intestines of numerous freshwater fish (e.g. Moravec 2001). Embryonated eggs (with Acanthor larvae) are excreted in the feces of the fish and ingested by the water isopod *Asellus aquaticus*. There they develop in the hemocoel into Acanthella larvae, which are infectious for the definitive host and are in turn ingested by fish by predation on the isopods (e.g. Andryuk 1974; Brattey 1986, 1988; Golvan 1969; Kennedy 1974; Lucius et al. 2018). The detection of this parasite species allows conclusions to be drawn about the American mink's diet and lifestyle. As the identified individuals of *A*. *lucii* were adults, it is likely that predation on infected freshwater fish, the definitive hosts of the parasite, must have taken place.

Analysis of the fecal samples revealed the presence of three parasites (Fig. 2a-c). Eggs of the fluke *A*. *alata* (Digenea) and the two hairworms *C*. *putorii* and *C*. *aerophila* (Nematoda) were identified. The Dunker's muscle fluke *A*. *alata* is distributed worldwide and parasitizes as an adult in the small intestine of carnivores. The main intermediate hosts in the life cycle are snail and amphibian species, but many other species of mammals, birds and reptiles can also be infected as paratenic hosts, as can humans as accidental hosts (e.g. Korpysa-Dzirba et al. 2021; Möhl et al. 2009). In contrast to *I*. *melis*, this Digenea species could not be identified during the examination of the organs, just as eggs of *I*. *melis* could not be detected in the feces. The eggs of *A*. *alata* and *I*. *melis* look very similar and are characterized by an ellipsoidal shape and the presence of an operculum (Dönges 1973; Zajac and Conboy 2012). They differ mainly in their size. The eggs identified in the fecal samples had an average size of 109.78 × 70 µm, which is within the range of already known egg sizes of *A*. *alata* (Möhl et al. 2009; Schmäschke 2014; Zajac and Conboy 2012). According to Dönges (1973), the eggs of *I*. *melis* reach a size between 142–149 × 83–91 µm and are thus larger than those of *A*. *alata*. It should be noted here that the morphology of *I*. *melis*, both in the egg stage and as an adult, is highly host-dependent (e.g. Dönges 1967, 1973; Nugaraitė et al. 2017). However, the eggs of *I*. *melis*, which have already been identified in *N*. *vison* were also between 115–125 × 80–95 in size and thus larger than those identified in this study (Hildebrand et al. 2015). This leads to the conclusion that the eggs identified here via MIFC belong to *A*. *alata*.

In addition, egg stages of the nematode *C*. *putorii* were detected in one fecal sample. This parasite species lives as an adultus in the stomach and small intestine of mustelids, cats and raccoons. The life cycle can be both direct and indirect, with earthworms being used as intermediate hosts. Infection can cause gastrointestinal symptoms such as vomiting (Deplazes et al. 2021). *Capillaria putorii* is already known as a parasite in American mink in Europe (e.g. Kołodziej‐Sobocińska et al. 2018; Kołodziej-Sobocińska et al. 2021; Martínez-Rondán et al. 2017; Nugaraitė et al. 2014, 2019; Shimalov and Shimalov 2001a; Torres et al. 2003, 2008), but this is the first time it's been found as a parasite of American mink in Germany.

The same applies to the lungworm *C*. *aerophila*, which was identified in the feces of one American mink. This nematode species parasitizes mainly in the respiratory tract of carnivores such as dogs and cats and can also infect humans. Infection occurs either through the ingestion of infective egg stages from the environment or through earthworms during ingestion, which can serve as facultative hosts (Deplazes et al. 2021; Traversa et al. 2011). *Capillaria aerophila* (syn. *Eucoleus aerophilus*) has already been described as a parasite for *N*. *vison* in Europe (e.g. Nugaraitė et al. 2014, 2019), but has not yet been detected in American mink populations from Germany until this study.

The different detection of parasite species at necropsy and fecal sample examination underlines the necessity of a combined parasitological processing of samples in order to obtain a good overview of the actual parasite diversity. Similar results are also shown in previous studies (e.g. Schantz et al. 2023).

In total, two tick species were identified as ectoparasites up to species level, as well as seven endoparasite species by examining the internal organs and the fecal samples. Compared to the possible parasite infestation in Europe (Tab. 2), only a few parasite species were identified in this study also with only low infestation numbers. Compared to earlier studies from Germany, the results for identified parasite species and prevalence also differ, especially for some digeneans and nematodes. The prevalence of ticks, cestodes and acanthocephalans, on the other hand, is more similar to that of our study (Christian 2010; Heddergott et al. 2016; Zschille et al. 2004). One reason for the differences could be the different locations of the study areas, another is that the animals examined in the other studies appear to have been examined directly, without prior freezing, which could possibly have an impact on the detection of parasites. In addition, the sample size could also be decisive. While Heddergott et al. (2016) examined 185 individuals of *N*. *vison* and Christian (2010) examined 121 individuals, our sample size is more comparable to Zschille et al. (2004) (n = 61 for ectoparasites, n = 66 for endoparasites). It should be mentioned that Heddergott et al. (2016) only examined the occurrence of cranial helminths and Christian (2010) the tick infestation, while the present study and Zschille et al. (2004) specialized in the occurrence of ectoparasites and parasites of the internal organs. The occurrence of parasites in newly spreading populations of invasive or alien species increases the longer the population is present in the area and becomes established (Kołodziej‐Sobocińska et al. 2018). As *N*. *vison* has been present in Germany since the 1950 s, it would be assumed that the prevalence and intensity of the parasite infestation is higher than proven in this study. However, it should also be considered that American mink continue to spread from the northern and eastern areas of Germany (Bonesi and Palazon 2007; Zschille et al. 2004) and establishment is therefore not yet complete in all areas. The results, especially the detection of two new parasite species for the American mink in Germany, show that a holistic approach of continuous studies on the parasite infestation of the American mink and other non-native species is necessary in order to better assess the potential threat they pose. This is also supported by studies from other European countries in which *N*. *vison* has been present for a longer period of time, which, in contrast to studies from Germany, have already been able to demonstrate some strong negative influences on native species and ecosystems (e.g. Aguiló-Gisbert et al. 2021, Nugaraité et al. 2019, Nordström et al. 2003). Finally, the seasonal factor should not be ignored, as this could be a possible reason for the low diversity of the parasite fauna. The animals examined originate from the autumn and winter months, when potential amphibian intermediate hosts of the digenean parasites (e.g. *A*. *alata*, *I*. *melis*) are no longer active. Kołodziej‐Sobocińska et al. (2018) also discovered seasonal differences in parasite infestation over a longer period of dispersal of the American mink and concluded that the abundance of nematodes decreases between September and June, while the numbers of digeneans generally increases during this period, but is highest in adult American mink between February and May, during the breeding season and therefore not in the period examined here.

### *Neogale vison* versus *Mustela lutreola*

The remaining western european populations of *M*. *lutreola* are located in northern Spain and also southern France, where the European mink lost half of its original range between 1980 and 2000 alone (Lodé et al. 2001; Maizeret et al. 2002; Maran et al. 2016). Both populations are assumed to be in decline and to consist of only a few hundred individuals (Maran et al. 2016). In Eastern Europe, the populations in the Danube Delta (Romania, Ukraine), particularly in Romania, are now considered to be the last remaining in situ populations of the European mink (Maran et al. 2016; Marinov et al. 2012). In Russia, population numbers at the end of the twentieth century were slightly higher than in the rest of Europe, but also declined rapidly, so that today there is no certainty about the actual size of existing populations (e.g. Maizeret et al. 2002; Maran et al. 2016; Skumatov and Saveljev 2006; Vada et al. 2023).

The possible drivers of decline are diverse and, in many countries, represent a combination of numerous influencing factors. In particular, habitat loss and hunting pressure (e.g. Lodé et al. 2001; Maran and Henttonen 1995; Maran 2003; Maran et al. 2016) are among the main reasons for the decline of *M*. *lutreola*. Nonetheless, there is also evidence that the American mink is contributing to the displacement of the European mink in some countries or preventing its reintroduction (e.g. Põdra et al. 2013).

**Fig. 3:**
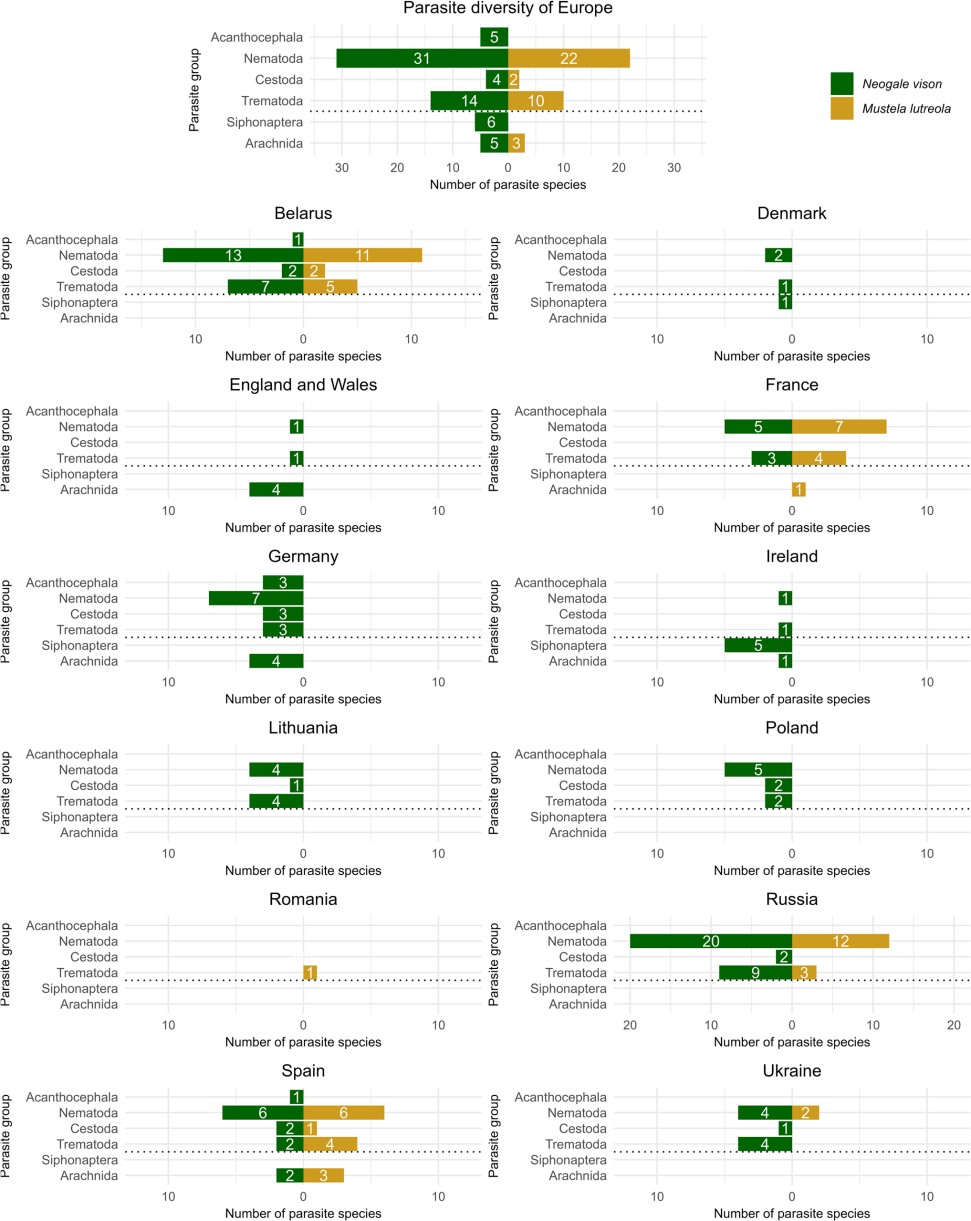
Number of documented parasite species per group in Europe and European countries; green = American mink (*Neogale vison*), yellow = European mink (*Mustela lutreola*)

Furthermore, the parasite load of the American mink could have an additional negative impact on the remaining populations of the European mink. Table 2 shows a comparison of ecto- and endoparasites that have been detected in both species in Europe to date. This shows that many parasite species can infest both the American and the European mink. 30 parasite species have so far been identified in both mustelid species in Europe, of which the nematode species *C*. *putorii*, *M*. *patens* and *Skrjabingylus nasicola* in particular occur in comparatively high prevalences (Fig. 3). *Neogale vison* has a much more diverse parasite fauna (Fig. 3, Tab. 2). It is also clear that the alien American mink is a competent host for native parasites, thus increasing the risk of transmission by spillback of parasites to native mustelids (Kelly et al. 2009). There are already 65 known parasite species of *N*. *vison* in twelve countries in Europe, while there is evidence of 37 parasite species of *M*. *lutreola* in six countries (Fig. 3, Tab. 2). Of course, it should be noted that there is less and older data available on the European mink (Tab. 2). Apart from the fact that the American mink already appears to be more competitive than *M*. *lutreola* due to its body size and higher adaptability, it also appears to carry a more diverse parasite fauna. The remaining populations of the European mink could therefore be exposed to a higher risk of infection with parasites and other pathogens introduced by *N*. *vison* into their newly developed areas due to the further spread of the American mink. Compared to the factors mentioned above, such as habitat loss, hunting and the occupation of ecological niches by the American mink, the risk of infection by parasites spread by *N*. *vison* appears to play a subordinate role for the remaining populations of *M*. *lutreola*. However, as the American mink continues to spread in the future and gradually establishes itself in new areas in Europe, this could become an additional problem for the remaining populations of *M*. *lutreola*, as these populations are already diminished to such an extent that infections with additional parasites could have devastating consequences for the European mink (Kelly et al. 2009). Other diseases for which *N*. *vison* is already known as a host, such as the Aleutian Mink Disease Virus, could also play an increasingly important role (e.g. Fournier-Chambrillon et al. 2004). The infection potential of the American mink may also be relevant for humans, as some of the pathogens it transmits have human pathogenic potential.

## Conclusion

Based on the parasite fauna identified in Germany, including two first detections, as well as the compilation of literature data, this study underlines the need of ongoing data collection. Continuous monitoring of the further spread of the American mink and the associated effects on native ecosystems (spread and transmission of diseases, predation, competition) is essential in order to be able to take the necessary protective measures at an early stage with regard to the risk of predation and infection as well as competition with other mustelids.

## Supplementary Information

Below is the link to the electronic supplementary material.

Supplementary file1 (DOCX 16.9 KB)

## Data Availability

No datasets were generated or analysed during the current study.
