# Supplementary material for: Metazoan parasite fauna of the American mink (Neogale vison) in comparison with the closely related European mink (Mustela lutreola) in Europe
Source: Parasitol Res. 2025 Aug 18;124(8):92. doi: 10.1007/s00436-025-08543-8 (PMC12361302; doi:10.1007/s00436-025-08543-8)
Supplement: Supplementary file 1 — Supplementary file1 (DOCX 16.9 KB) [file 436_2025_8543_MOESM1_ESM.docx]

|  | **Male + Female (M+F) n=50** | | | **Male (M) n=36** | | | **Female (F) n=14** | | | |
| --- | --- | --- | --- | --- | --- | --- | --- | --- | --- | --- |
|  | Min. - Max | Mean | S.D. (±) | Min. - Max | Mean | S.D. (±) | Min. - Max | Mean | S.D. (±) |  |
| **Total weight [g] *** | 594.6 - 2127.8 | 1163.3 | 340.7 | 594.6 - 2127.8 | 1320.2 | 265.4 | 600.0 - 900.0 | 759.9 | 86.8 |  |
| **Carcass weight [g] *** | 500.0 - 1837.0 | 983.7 | 301.6 | 511.9 - 1837.0 | 1123.3 | 231.5 | 500.0 - 750.0 | 624.9 | 90.8 |  |
| **Standard length [cm] *** | 33.5 - 48.4 | 39.5 | 4.0 | 34.0 - 48.4 | 41.2 | 3.3 | 33.5 - 39.0 | 35.3 | 1.7 |  |
| **Tail length [cm] *** | 12.0 - 24.0 | 20.1 | 2.4 | 12.0 - 24.0 | 20.9 | 2.2 | 15.0 - 20.5 | 18.1 | 1.6 |  |
| **Total length [cm] *** | 46.0 - 70.9 | 59.6 | 5.7 | 46.0 - 70.9 | 62.1 | 4.8 | 49.0 - 56.0 | 53.4 | 1.9 |  |
| **Hind foot length [cm] *** | 4.5 - 7.42 | 6.2 | 0.7 | 4.97 - 7.42 | 6.5 | 0.5 | 4.5 - 6.02 | 5.5 | 0.4 |  |
| **Ear length [cm]** | 0.8 - 2.86 | 1.9 | 0.7 | 0.9 - 2.86 | 2.0 | 0.7 | 0.8 - 2.57 | 1.7 | 0.7 |  |

* Males being significantly different from females (α=0.05, Bonferroni-adjusted)

Metazoan parasite fauna of the American mink (*Neogale vison*) in comparison with the closely related European mink (*Mustela lutreola*) in Europe

Parasitology Research

Anna V. Schantz, Robin Stutz, Anne Steinhoff, Norbert Peter, Sven Klimpel

*Corresponding author: schantz@bio.uni-frankfurt.de

Institute for Ecology, Evolution and Diversity, Goethe-University, Max-von-Laue-Str. 13, Frankfurt/Main D-60438, Germany
